# The Predictive Validity of the Structured Assessment of Violence Risk in Youth for Young Spanish Offenders

**DOI:** 10.3389/fpsyg.2017.00577

**Published:** 2017-04-12

**Authors:** Elena Ortega-Campos, Juan García-García, Flor Zaldívar-Basurto

**Affiliations:** Standing Seminar on Juvenile Justice, Psychology Department, University of AlmeríaAlmería, Spain

**Keywords:** risk assessment, SAVRY, predictive validity, recidivism, younger offenders

## Abstract

The present study examined the predictive validity of the Structured Assessment of Violence Risk in Youth (SAVRY) in a group of young Spanish offenders. The sample is made up of 594 minors from the Juvenile Court, between the ages of 14 and 18 at the time they committed the delinquent act. The SAVRY was able to differentiate between low and high-risk younger offenders. Mean scores on risk factor are greater in the group of recidivist offenders, the group of non-recidivist shows higher mean scores in Protective domain. The accuracy of the instrument is high (AUC_RiskTotalScore_ = 0.737 and AUC_SummaryRiskRating_ = 0.748). An approximation of the predictive validity study of the SAVRY in Spanish younger offenders is presented. The results obtained support the SAVRY good functioning with not English samples.

## Introduction

Interest in risk assessment of juvenile offenders has triggered the development of numerous instruments specifically designed for young offenders (Hoge and Andrews, 2001, 2003; Borum et al., 2002, 2003, 2006; Stockdale, 2008; McKinlay et al., 2015). Based on principles of the Risk-Need-Responsivity (RNR) model, instruments for measuring risk in juvenile offenders are used in Juvenile Justice in order to identify those juvenile offenders who need intervention (risk), the criminogenic needs they present (needs) and the strategies that should be used with these young offenders (responsivity) (Andrews et al., 1990; Andrews and Bonta, 2010; Polaschek, 2012; Childs et al., 2014).

The aim of such risk instruments is to help with decisions about what measures should be taken with each juvenile offender (Vincent et al., 2011; Childs et al., 2013). Interventions based on the criminogenic needs of the juvenile offender are more effective than general interventions (Andrews and Bonta, 2010).

The instruments currently in use look for the presence of factors that increase or decrease the likelihood of the offender carrying out another sanctionable antisocial behavior (S-ASB). A risk factor for S-ASB is a variable that predicts a high probability of recidivism, by contrast, protective factors present a lower probability of recidivism, in addition to mitigating the effect of risk factors (Farrington et al., 2012).

Research on recidivism in juvenile justice has made great efforts to identify risk factors that young offenders present, with the understanding that their elimination would reduce S-ASBs (Andrews and Bonta, 2010). Factors that show the strongest predictive associations for repeat S-ASBs are: prior S-ASBs (Cuervo and Villanueva, 2015), age when the first delinquent act was committed (Van der Put, 2011), problems at school or work (Viljoen et al., 2009; Weerman, 2010; Van der Put, 2011; Cuervo and Villanueva, 2015; Harder et al., 2015), antisocial peers (Fergusson et al., 2007; Van der Put, 2011; Cuervo and Villanueva, 2015; Harder et al., 2015; Ortega-Campos et al., 2016; Makarios et al., forthcoming), poor use of leisure time (Van der Put, 2011; Cuervo and Villanueva, 2015; Harder et al., 2015), antisocial personality/behavior (Cuervo and Villanueva, 2015; Makarios et al., forthcoming), lack of parental supervision (Chambers et al., 2001; Álvarez-García et al., 2016; Ortega-Campos et al., 2016; Makarios et al., forthcoming) and criminality in family members (Murray and Farrington, 2008; Geller et al., 2009). There are other holistic approaches in juvenile offenders (e.g., the Good Lives Model) premised on the idea that the offenders need to build capabilities and strengths, in order to reduce their risk of reoffending (Cipolletta, 2011; Dumas and Ward, 2016).

This study has used the Structured Assessment of Violence Risk in Youth (SAVRY) in order to predict risk in young offenders (Borum et al., 2002, 2003). The SAVRY was developed for use by professionals who conduct assessments and interventions with youth (Borum et al., 2006). The SAVRY includes items that measure historical, social/contextual, and individual risk factors, and protective factors. Each of these factors have been found to be empirically related to violence and delinquency (e.g., Childs et al., 2014).

The SAVRY has been adapted to different countries, including the USA (Chapman et al., 2006), Finland (Gammelgard et al., 2008), Netherlands (Duits et al., 2008), UK (Dolan and Rennie, 2008), Germany (Klein et al., 2012), Australia (Shepherd et al., 2014a), Singapore (Chu et al., 2016), China (Zhou et al., 2017) and Spain (Hilterman et al., 2014, 2016). In all these studies the SAVRY shows adequate psychometric properties (Dolan and Rennie, 2008; Duits et al., 2008; Gammelgard et al., 2008; Klein et al., 2012; Hilterman et al., 2014; Chu et al., 2016).

Before using an instrument, it is essential to know that it has a good functioning in the population to which is directed. In Spain, the study of the predictive validity of SAVRY was performed with a sample of 145 subjects who were serving a probation measure in a region of Spain (Hilterman et al., 2014). Although the first approximation to the study of SAVRY functioning in Spanish context is adequate, a broader study using a larger sample and including children that are not complying with a single judicial measure is necessary. Therefore, the aim of the present study is to examine the predictive validity SAVRY in juvenile offenders in Spain.

## Materials and Methods

### Participants

The study sample was made up of the set of juveniles who were charged in a court case in Spain during a year of study. Their first case opened during the period of study is taken as the baseline incident. The juveniles included in this study had committed some S-ASB specified in the Spanish Penal Code. Any juvenile who commits an S-ASB will be judged under Organic Law 5/2000 if at the time of the act he/she was between the ages of 14 and 18. The sample was composed of a total of 594 juveniles. With regard to sociodemographic variables, 85.4% of the sample are male and 79% are Spanish nationals. At the time of the S-ASBs analyzed here, 45.1% of the offenders were 14–15 years old, age at which 57.4% of the study sample committed their first S-ASB. The average age of the sample at time of the S-ASBs studied was 15.63 years (*SD* = 1.08, range = 14–17).

### Measure

The Spanish adaptation (Vallés and Hilterman, 2006) of the SAVRY (Borum et al., 2002, 2003) was used in this study. The SAVRY is designed to predict violent behavior in youth between 12 and 18 years of age. The SAVRY consists of 30 items, grouped into 3 risk domains (historical, social/contextual and individual) and one protective domain. There are 24 risk items and 6 protective items. Each risk factor can be scored at three levels: low (0 point), moderate (1 point) and high (2 points). Protective factors allow two levels of response: present (1 point) or absent (0 point). The SAVRY provides a total risk level of recidivism for each juvenile offender, Risk Total Score (RTS) and summary risk rating (SRR) (Borum et al., 2006).

In Spain, the SAVRY has been used in the Catalonian Juvenile Justice system, showing adequate reliability coefficients for both global scores of the instrument (ICC_RTS_ = 0.79, ICC_SRR_ = 0.66 and α = 0.90), and for partial scores (ICC_Historical_ = 0.89, ICC_Social_ = 0.60, ICC_Individual_ = 0.61, ICC_Protective_ = 0.67, α_Historical_ = 0.77, α_Social_ = 0.72, α_Individual_ = 0.83 and α_Protective_ = 0.78) (Hilterman et al., 2014, 2016).

### Recidivism

For this study, there is recidivism of S-ASB when the juvenile is charged in a new court case in the Juvenile Court of Almeria (Spain) at some time after the baseline case established as point of reference. The recidivism study covered a period of 2 years from the date of each offender’s baseline court case (Cuervo and Villanueva, 2015). In this study, 35.5% of the sample was recidivist.

### Procedure

The Human Bioethics Committee of the University of Almeria approved the present study. The data were extracted anonymously from the youth court records with the permission of the juvenile court of Almería and none of the researchers had access to the juveniles personal data.

### Data Analysis

Internal consistency was examined using Cronbach’s α, within its range of 0 to 1.0, values of 0.70 and above considered appropriate (DeVellis, 2011).

The χ^2^ statistic was used as a test of statistical significance (Tabachnick and Fidell, 2007) for the categorical variables. For the metric variables, after checking for normality with Kolmogorov’s test, Mann–Whitney’s non-parametric test was calculated (Fay and Proschan, 2010).

In the tests of statistical significance, an effect size index is calculated when the results are statistically significant. The effect size indices report the real importance of the results obtained from the significance tests (Vacha-Haase et al., 2000; American Psychological Association, 2009; García et al., 2011).

As a complement to the study of validity evidence for the SAVRY, the receiver operating characteristic curve (ROC) was calculated. ROC analysis expresses predictive validity in the area under the curve (AUC), which can range from 0.0 (perfect negative prediction) to 1 (perfect positive prediction). An AUC of 0.50 corresponds to a prediction that is no better than chance. AUCs between 0.56 and 0.64 are considered small effects, AUCs above 0.64 are medium effects, and AUCs greater than 0.71 are described as large effects (Rice and Harris, 2005).

## Results

**Table 1** presents the descriptive statistics of the SAVRY domains (historical, social, individual and protective) and global scores. Mean score were 6.70 for the RTS and 8.59 for the SRR. Mean scores for the SAVRY risk and protective factors were 3.51 for the historical domain, 2.97 for the individual domain, 2.12 for the social domain and 2.99 for the protective domain.

**Table 1:** Structured Assessment of Violence Risk in Youth (SAVRY) descriptive statistics and Spearman’s rho correlation coefficient.

	*M*(*SD*)	Range	SAVRY_Historical_	SAVRY_Social_	SAVRY_Individual_	SAVRY_Protective_	RTS
SAVRY_Historical_	3.51 (3.26)	0–16					
SAVRY_Social_	2.12 (2.37)	0–12	0.68^∗^				
SAVRY_Individual_	2.97 (2.72)	0–13	0.69^∗^	0.71^∗^			
SAVRY_Protective_	2.99 (1.88)	0–6	-0.66^∗^	-0.73^∗^	-0.79^∗^		
RTS	6.70 (7.84)	0–36	0.82^∗^	0.87^∗^	0.89^∗^	-0.87^∗^	
SRR	8.59 (7.40)	0–36	0.88^∗^	0.87^∗^	0.90^∗^	-0.81^∗^	0.97^∗^


*RTS, Risk Total Score; SRR, summary risk rating. *N* = 594; ^∗^*p* < 0.01.*

The correlations between the factors and global scores on the SAVRY are statistically significant (*p* < 0.01) and large in magnitude. Correlations between the RTS score and factors of the SAVRY are statistically significant with values between 0.82 and 0.89. Correlations between the SRR score and factors of the SAVRY are statistically significant and greater than 0.81. Finally, the correlation between the SAVRY totals (RTS and SRR) is 0.97.

**FIGURE 1:**
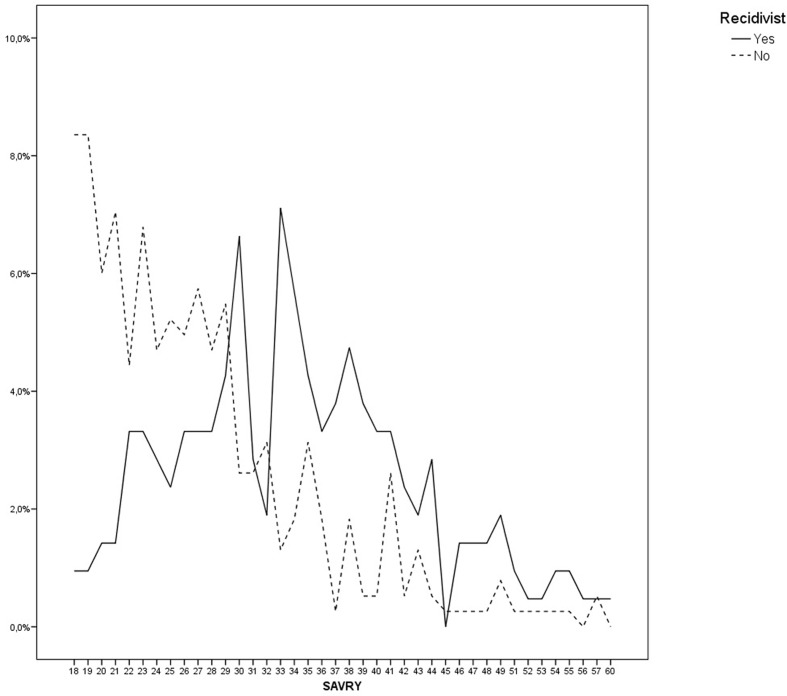
**Structured Assessment of Violence Risk in Youth (SAVRY) scores for the younger offenders recidivist and non-recidivist**.

**Figure 1** presents the percentage of younger offenders in relation to the scores obtained in the SAVRY, the global scores obtained from the group of younger offenders non-recidivist is compared with the group of youngers recidivist. As it can be noted, there are some differences in the younger scores, non-recidivist present low risk scores and recidivist present high risk in the SAVRY.

Cronbach’s α was calculated in order to study the internal consistency of the SAVRY instrument. For the SRR score, the Cronbach’s α coefficient presents a value of 0.89, 95%CI [0.88,0.90]. Reliability coefficients were calculated for the risk domains of the SAVRY, for historical domain α = 0.79, 95%CI [0.77,0.82], for social domain α = 0.68, 95%CI [0.64,0.72], for individual domain α = 0.76, 95%CI [0.73,0.79] and for protective domain α = 0.81, 95%CI [0.79,0.84].

To study the discriminant validity evidence, a comparison of the SAVRY scores according to recidivism in committing a S-ASB was calculated. The instrument’s total and partial scores do not show normality, **Table 2** presents the mean values and standard deviation for the youths’ total and partial scores on the SAVRY according to their recidivism in S-ASB, the Mann–Whitney *U* test, significance and estimated effect size. All comparisons are statistically significant. Estimated effect sizes are greater than 0.34 in all cases (**Table 2**).

**Table 2:** Recidivism and SAVRY.

		*M*(*SD*)	*Z*	*r* (ES)
SAVRY_Historical_	Recidivism	4.84(3.50)	-8.682*	0.35
	Non-recidivism	2.77 (2.87)		
SAVRY_Social_	Recidivism	3.14(2.50)	-8.407*	0.34
	Non-recidivism	1.55 (2.09)		
SAVRY_Individual_	Recidivism	4.39(2.71)	-10.081*	0.41
	Non-recidivism	2.19 (2.40)		
SAVRY_Protective_	Recidivism	2.11(1.61)	-8.531*	0.35
	Non-recidivism	3.47 (1.84)		
Risk Total Score	Recidivism	10.57(8.24)	-9.763*	0.39
	Non-recidivism	4.56 (6.72)		
Summary risk rating	Recidivism	12.38(7.45)	-10.020*	0.40
	Non-recidivism	6.51 (6.50)		


*^∗^*p* < 0.01.*

In order to understand the behavior of the SAVRY items, the mean score, standard deviation and corrected item-total correlation for each item have been calculated. The risk items of Poor School Achievement and Low Interest/Commitment to School or Work present mean scores greater than 1. In the Protective domain, items that are most notably absent in the young offenders are Strong Commitment to School or Work and Resilient Personality Traits. Coefficients for the corrected item-total correlations are positive and different from zero, with values greater than 0.33, except for the items History of Self-Harm or Suicide Attempts (*r* = 0.17) and Peer Rejection (*r* = 0.33) (**Table 3**).

**Table 3:** Percentage of recidivism and SAVRY items and SAVRY item discrimination index.

	Total (%)	Non-R (%)	R (%)	*p*	ES (*r*)	Corrected
	*N* = 594	*N* = 383	*N* = 211			item-total
						correlation
**SAVRY_Historical_**						
History of violence	75.3	81.7	63.5	<0.01	0.20	0.57
History of non-violent offending	82.7	88.0	73.0	<0.01	0.19	0.47
Early initiation of violence	85.0	91.1	73.9	<0.01	0.23	0.61
Past supervision/intervention failures	80.0	86.9	67.3	<0.01	0.24	0.67
History of self-harm or suicide attempts	98.3	98.7	97.6	0.628		0.17
Exposure to violence in the home	85.2	89.6	77.3	<0.01	0.16	0.51
Childhood history of maltreatment	93.3	95.8	88.6	<0.01	0.13	0.39
Parental/caregiver criminality	85.4	91.4	74.4	<0.01	0.23	0.51
Early caregiver disruption	68.2	72.8	59.7	<0.01	0.13	0.40
Poor school achievement	16.3	22.5	5.2	<0.01	0.29	0.46
**SAVRY_Social/Contextual_**						
Peer delinquency	58.4	70.5	36.5	<0.01	0.34	0.62
Peer rejection	86.0	89.0	80.6	0.01	0.12	0.20
Stress and poor coping	81.8	86.7	73.0	<0.01	0.17	0.33
Poor parental management	59.9	69.2	43.1	<0.01	0.27	0.66
Lack of personal/social support	86.0	88.0	82.5	0.125		0.46
Community disorganization	78.8	84.1	69.2	<0.01	0.17	0.49
**SAVRY_Individual_**						
Negative attitudes	73.1	82.0	56.9	<0.01	0.27	0.61
Risk-taking/impulsivity	60.3	69.2	44.1	<0.01	0.25	0.54
Substance use difficulties	88.7	93.5	80.1	<0.01	0.20	0.38
Anger management problems	80.8	88.3	67.3	<0.01	0.25	0.52
Low empathy/remorse	92.6	96.6	85.3	<0.01	0.22	0.37
Attention-deficit/hyperactivity disorder	84.8	89.8	75.8	<0.01	0.19	0.36
Poor compliance	69.9	79.9	51.7	<0.01	0.29	0.52
Low interest/commitment to school or work	30.0	39.2	13.3	<0.01	0.34	0.57
**SAVRY_Protective_**						
Prosocial involvement	42.3	53.5	21.8	<0.01	0.30	0.64
Strong social support	84.0	87.7	77.3	<0.01	0.13	0.57
Strong attachments and bonds	77.1	83.8	64.9	<0.01	0.21	0.64
Positive attitude toward intervention and authority	50.3	61.9	29.4	<0.01	0.31	0.62
Strong commitment to school or work	32.7	43.1	13.7	<0.01	0.29	0.59
Resilient personality traits	12.5	17.2	3.8	<0.01	0.19	0.37


In order to study which items made the greatest contribution to higher levels of risk in the youths studied, **Table 3** presents a between-group comparison (recidivism/non-recidivism) of the values of the significance test and the estimated effect size. As additional information, the percentage of youths who show a low level on each SAVRY item is presented for the total group, for the group of recidivism and non-recidivism.

In the between-group analysis (recidivism/non-recidivism) of the SAVRY items, statistically significant differences are found on all items, with the exceptions of History of Self-Harm or Suicide Attempts and Lack of Personal/Social Support.

**FIGURE 2:**
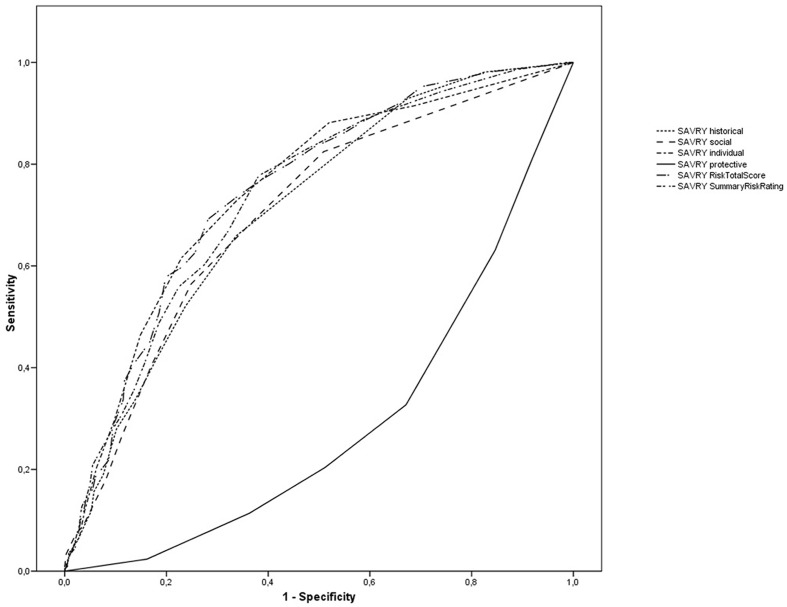
**Receiver operating characteristic (ROC) for the SAVRY scores**.

In order to study the predictive validity evidence for the SAVRY, ROC curves were calculated. **Table 4** presents the correlation coefficient for total and partial scores on the SAVRY with recidivism in S-ASB, the AUCs, and confidence interval at 95%. Correlations between SAVRY scores and repeated S-ASB are statistically significant. AUCs are statistically significant, presenting values greater than 0.70, except for the Protective factors (**Figure 2**).

**Table 4:** Spearman’s rho correlation coefficient and area under the curve values of SAVRY for recidivism outcomes.

	*r*	AUC(SE)	CI 95%
SAVRY_Historical_	0.357^∗^	0.711(0.021)^∗^	[0.669,0.753]
SAVRY_Social_	0.345^∗^	0.702(0.022)^∗^	[0.658,0.745]
SAVRY_Individual_	0.414^∗^	0.747(0.021)^∗^	[0.706,0.788]
SAVRY_Protective_	-0.350^∗^	0.291(0.022)^∗^	[0.249,0.334]
Risk Total Score	0.401^∗^	0.737(0.021)^∗^	[0.695,0.779]
Summary risk rating	0.411^∗^	0.748(0.020)^∗^	[0.706,0.788]


*^∗^*p* < 0.01.*

## Discussion

This study presents an examination of the predictive validity of the SAVRY in its application to a sample of young offenders from the Juvenile Court of Spain. It represents another step forward in the study of instruments for predicting risk of recidivism. In particular, this study of the SAVRY supplies information that can be compared to other studies performed in this country and internationally (McEachran, 2001; Catchpole and Gretton, 2003; Borum et al., 2006; Dolan and Rennie, 2008; Duits et al., 2008; Gammelgard et al., 2008; Lodewijks et al., 2010; Vincent et al., 2011; Klein et al., 2012; Childs et al., 2013, 2014; Hilterman et al., 2014, 2016; Shepherd et al., 2014a; Chu et al., 2016), as well as information for carrying out systematic, meta-analysis reviews (Schwalbe, 2007, 2008; Olver et al., 2009; Fazel et al., 2012; Singh et al., 2013), and for future generalization studies focusing on the instrument’s reliability and validity.

Alpha coefficients have been calculated for the SAVRY scores. Our results agree with those of prior studies (Welsh et al., 2008; Klein et al., 2012; Childs et al., 2014; Hilterman et al., 2014, 2016; Shepherd et al., 2014b). When comparing total and partial mean scores on the SAVRY instrument, the scores from this study are lower than those calculated in other research studies (Dolan and Rennie, 2008; Gammelgard et al., 2008; Lodewijks et al., 2008; Meyers and Schmidt, 2008; Hilterman et al., 2014, 2016; Shepherd et al., 2014a; Chu et al., 2016). This difference may be due to the sample used in each case. In studies with young offenders, the sample is usually made up of juveniles who have been subject to some kind of disciplinary measure from Juvenile Justice. For this study, the sample selection criterion was not the application of a disciplinary measure, we selected the total set of juveniles for whom a court case had been opened in the Juvenile Court during the period studied, making our sample more heterogeneous, possibly presenting lower levels of risk.

The items that make up the SAVRY instrument represent factors that the literature has proven to be related to young offenders’ recidivism in S-ASB. Regarding the individual items, the total group of minors obtained higher scores on the following: Poor School Achievement and Low Interest/Commitment to School or Work (Viljoen et al., 2009; Weerman, 2010; Van der Put, 2011; Cuervo and Villanueva, 2015; Harder et al., 2015), Peer Delinquency (Fergusson et al., 2007; Van der Put, 2011; Cuervo and Villanueva, 2015; Harder et al., 2015; Ortega-Campos et al., 2016; Makarios et al., forthcoming) and Poor Parental Management (Chambers et al., 2001; Álvarez-García et al., 2016; Ortega-Campos et al., 2016; Makarios et al., forthcoming). The lowest mean score in the group of protective factors was for Resilient Personality Traits (Mowder et al., 2010). The SAVRY items with the greatest estimated effect size were the factors from the RNR model (Andrews et al., 1990) as predictors of S-ASB: History of Antisocial Behavior, Antisocial Personality Pattern, Antisocial Peers, School and/or Employment Conflicts, Family Support and Substance Abuse (Andrews and Bonta, 2010).

To study the instrument’s reliability, the corrected item-total correlation coefficient for each item was calculated. These correlations were different from zero, positive and statistically significant for all items, indicating that no item should be eliminated from the instrument (Crocker and Algina, 1986; Abad et al., 2011).

Statistically significant correlations were obtained between total and partial scores on the SAVRY. All correlations were greater than 0.66, with inverse (negative) correlations in the case of the protective factors. In studying the SAVRY’s validity, between-group analyses were carried out (repeat offenders vs. non-repeaters) for each of the total and partial scores. Statistically significant differences were found between the repeat offenders and the non-repeaters for all comparisons made, effect sizes were medium, greater than 0.34. The group of repeat offenders obtained higher mean scores for the global measures (SRR and RTS) and for the risk factors. The group of non-repeaters showed higher scores in the protective factors.

The fact that the recidivist offenders showed risk factors in greater number and strength, and a reduced presence of protective factors, supports the idea proposed in the RNR model (Andrews and Bonta, 2010). The RNR model postulates the existence of risk and protective factors in relation to recurrence of S-ASB, and that youngers recidivist present greater levels of risk factors. Consequently, interventions from Juvenile Justice should address the criminogenic needs that the juvenile presents, in order for the intervention and the youth’s experience with Juvenile Justice to be as effective as possible, and to meet the objective of reducing the effect and presence of risk factors in the repeat offenders (Andrews and Bonta, 2010; Polaschek, 2012; Childs et al., 2014; Ortega-Campos et al., 2016). The SAVRY proves to be a good instrument for younger offenders, discriminating level of risk and risk factors between recidivist and non-recidivist (McEachran, 2001; Catchpole and Gretton, 2003; Gammelgard et al., 2008; Schwalbe, 2008; Welsh et al., 2008; Viljoen et al., 2009; Vincent et al., 2011; Hilterman et al., 2014).

Mean scores on risk factors are greater in the group of recidivist offenders; the group of non-recidivists shows higher mean scores in the Protective factors (Shepherd et al., 2014b). This finding supports research which indicates that positive factors are crucial in the desistance from recidivism for young offenders (Stouthamer-Loeber et al., 2004; Lodewijks et al., 2010; Shepherd et al., 2014b). The items included in the SAVRY correspond to variables related to recurrence of S-ASB in juveniles, according to the scientific literature (Borum et al., 2002, 2003, 2006). The items that show relevant differences and higher values of effect size in the comparison between the younger offenders and the younger recidivist are in the historical domain: History of Violence (Cuervo and Villanueva, 2015), Early Initiation of Violence (Van der Put, 2011; Ortega-Campos et al., 2016), Parental/Caregiver Criminality (Murray and Farrington, 2008; Geller et al., 2009) and Poor School Achievement. In the social domain, Peer Delinquency (Fergusson et al., 2007; Van der Put, 2011; Cuervo and Villanueva, 2015; Harder et al., 2015; Ortega-Campos et al., 2016; Makarios et al., forthcoming) and Poor Parental Management (Chambers et al., 2001; Álvarez-García et al., 2016; Ortega-Campos et al., 2016; Makarios et al., forthcoming). Finally, in the individual domain, Negative Attitudes (Cuervo and Villanueva, 2015; Makarios et al., forthcoming) and Low Interest/Commitment to School or Work (Viljoen et al., 2009; Weerman, 2010; Van der Put, 2011; Cuervo and Villanueva, 2015; Harder et al., 2015).

The ROC analysis is especially indicated in cases where a decision must be made, where it is essential to know in detail how accurate the different diagnostic tests are, and whether they correctly classify patients in categories or conditions related to a certain criterion (Fazel, 2013; Mossman, 2013). The total and partial scores of the instrument present statistically significant correlations different from zero and greater than 0.34 between the scores and factors of the SAVRY and recurrence of the S-ASB. The AUC values are statistically significant, and greater than 0.70 (Lodewijks et al., 2008; Schmidt et al., 2011; Hilterman et al., 2014, 2016), except for the protective factors which obtained a value of 0.29. In this study, we have taken direct scores for the protective factors, since they are not a risk factor for predicting recurrence of the S-ASB. The ROC and correlational analyses revealed that the SAVRY total and partial scores are moderately predictive juvenile recidivism (Dolan and Rennie, 2008; Gammelgard et al., 2008; Lodewijks et al., 2008; McGowan et al., 2011; Schmidt et al., 2011; Hilterman et al., 2014; Shepherd et al., 2014a; Chu et al., 2016). In addition, the SAVRY presents similar predictive values than others risk assessment instruments (Chu et al., 2016).

The SAVRY has demonstrated a good predictive capacity of juvenile offenders recidivism in medium term, similar to that found in other studies. It has also been shown to discriminate between youngers with low and high risk of recidivism (Dolan and Rennie, 2008; Gammelgard et al., 2008; Klein et al., 2012; Chu et al., 2016). Overall, the SAVRY is well adapted to the Spanish context. The use of instruments to predict recidivism in Juvenile Justice is a very usefull tool in the process of decision making, as it takes the actual risk and protection factors that the younger presents as references. Such a tool is important in the creation and adaptation of the interventions that are performed with the youngers, ensuring that the will be done to maximize the benefit for them. The instruments for predicting recidivism risk have a double objective: in the short term they aim to identify the level of risk and of youngers, identifying the risks and protection factors; in the medium term they aim to create prevention and intervention plans and programs with youngers based on the risk and protection factors presented by each younger (Dumas and Ward, 2016; Ward and Fortune, 2016).

In the present study an approximation of the predictive validity of the SAVRY in Spanish younger offenders is presented. The results obtained support the knowledge of how the SAVRY functions with not English samples.

## Author Contributions

Conceived and designed the experiments: EO-C, JG-G, and FZ-B. Performed the experiments: EO-C. Analyzed the data: EO-C and JG-G. Wrote the paper: EO-C, JG-G, and FZ-B.

## Conflict of Interest Statement

The authors declare that the research was conducted in the absence of any commercial or financial relationships that could be construed as a potential conflict of interest.
